# Genome annotation improvements from cross-phyla proteogenomics and time-of-day differences in malaria mosquito proteins using untargeted quantitative proteomics

**DOI:** 10.1371/journal.pone.0220225

**Published:** 2019-07-29

**Authors:** Lisa Imrie, Thierry Le Bihan, Áine O'Toole, Paul V. Hickner, W. Augustine Dunn, Benjamin Weise, Samuel S. C. Rund

**Affiliations:** 1 SynthSys–Synthetic and Systems Biology, School of Biological Sciences, University of Edinburgh, Edinburgh, United Kingdom; 2 Centre for Immunity, Infection and Evolution, University of Edinburgh, Edinburgh, United Kingdom; 3 Rapid Novor, Kitchener, Ontario, Canada; 4 Institute of Evolutionary Biology, University of Edinburgh, Edinburgh, United Kingdom; 5 Eck Institute for Global Health, University of Notre Dame, Notre Dame, Indiana, United States of America; 6 Boston Children's Hospital, Boston, Massachusetts, United States of America; University of California-Davis, UNITED STATES

## Abstract

The malaria mosquito, *Anopheles stephensi*, and other mosquitoes modulate their biology to match the time-of-day. In the present work, we used a non-hypothesis driven approach (untargeted proteomics) to identify proteins in mosquito tissue, and then quantified the relative abundance of the identified proteins from *An*. *stephensi* bodies. Using these quantified protein levels, we then analyzed the data for proteins that were only detectable at certain times-of-the day, highlighting the need to consider time-of-day in experimental design. Further, we extended our time-of-day analysis to look for proteins which cycle in a rhythmic 24-hour (“circadian”) manner, identifying 31 rhythmic proteins. Finally, to maximize the utility of our data, we performed a proteogenomic analysis to improve the genome annotation of *An*. *stephensi*. We compare peptides that were detected using mass spectrometry but are ‘missing’ from the *An*. *stephensi* predicted proteome, to reference proteomes from 38 other primarily human disease vector species. We found 239 such peptide matches and reveal that genome annotation can be improved using proteogenomic analysis from taxonomically diverse reference proteomes. Examination of ‘missing’ peptides revealed reading frame errors, errors in gene-calling, overlapping gene models, and suspected gaps in the genome assembly.

## Introduction

*Anopheles stephensi* is a major malaria vector in southern Asia where its geographic range extends across the Indian subcontinent [1]. Research on the African *Anopheles gambiae* mosquito has demonstrated that the behavior and physiology of the mosquito is highly dependent on circadian biology and time-of-day. For example, ~20% of *An*. *gambiae* genes were rhythmically expressed over the 24-hour day [2]; rhythmically expressed mosquito olfaction genes correspond with rhythmic proteins levels and time-of-day changes in electrophysiological sensitivity to host odorants [3]; and time-of-day effects are associated with mosquito insecticidal resistance[4]. *An*. *stephensi* has been demonstrated to have 24-hour nocturnal rhythms of flight behavior that persists even in the absence of light:dark cues [5]. Finally, rhythms in the biology of the mosquito, and indeed possibly in the human host and *plasmodium* parasite, may interact to affect disease transmission [6–8].

To date, the genomes of two strains of *An*. *stephensi* have been sequenced, one from India and one from Pakistan (SDA-500) [9, 10]. To our knowledge, proteomics in this species is limited to an Edman degradation of their salivary glands [11]; mass spectrometry proteomics analysis of salivary proteomes [11, 12]; fat bodies [13, 14]; midguts/fat bodies [14]; a mass spectrometry proteomics analysis of ageing in the head and thorax [15]; and a recent work across multiple tissues which included genome annotation improvements [16].

In *An*. *gambiae*, several mass spectrometry-based studies have been performed on various tissues, including the antennae, head, body, midgut peritrophic matrix, salivary glands, and cuticle [3, 17–20]. Proteomic experiments can be used to identify post-translational modification, improve genome annotation, and to identify and quantify proteins in a biological sample [16, 21, 22].

A previous study in *An*. *gambiae* mosquito antennae utilized targeted quantitative proteomics, in which the mass spectrometer was tuned to specifically identify and quantify the protein abundance of proteins from an *a priori* list of genes of interest [3] where only targeted proteins are interrogated. Targeted proteomics is a powerful technique, allowing the verification of a defined working hypothesis on specific proteins that are quantified.

In the present work, we used a non-hypothesis driven approach (untargeted proteomics) to identify proteins in mosquito tissue. In addition, we quantified the relative abundance of the identified proteins from *An*. *stephensi* bodies. Such an untargeted, label free, quantitative analysis has been used on diverse tissues such as mammalian cells, yeast, bacteria, and *Ostreococcus tauri* algae [23–26]. Using these quantified protein levels, we then analyzed the data for proteins that were only detectable at certain times-of-the day, highlighting the need to consider time-of-day in experimental design. Further, we extended our time-of-day analysis to look for genes that are not only detectable at certain times-of-day, but which cycle in a rhythmic 24-hour (“circadian”) manner.

Annotation of the *An*. *stephensi* genome is far less complete than that of the model mosquito, *An*. *gambiae*. Proteogenomic analysis can be used to improve these annotations, particularly by experimentally validating computationally-derived open reading frame (ORF) predictions [27]. Additionally, proteogenomic analysis can be used in the identification of variant sequences and novel splicing sites [28, 29]. In these analyses, peptides detected using mass spectrometry were compared with reference proteomes. Peptide sequences not found in predicted protein coding regions indicate mis-annotations such as missing exons or entire missing genes, while peptide sequences with single amino-acid differences between the experimentally detected sequence and reference proteome may represent sequencing errors or polymorphisms. Here we apply proteogenomic analysis to *An*. *stephensi*, but employing a novel protocol, we reference over 30 predicted proteomes from other vector species to search against our experimentally derived peptides. We reveal that genome annotation can be improved using proteogenomic analysis from taxonomically diverse reference proteomes.

## Results and discussion

### Global survey of the proteome without fractionation

Using an untargeted proteomics approach without any fractionation on pooled samples of 10 whole mosquitoes harvested across the 24hr day, we have identified 12641 unique peptides (having a Maxquant score of 45 and more) mapping to ~1700 (identified with at least 2 peptides) *An*. *stephensi* proteins (S1 Table). *An*. *stephensi* is thought to have ~11,789 genes [9] thus, with no fractionation we observed 13% of the predicted proteome. Future studies could increase the number of detectable proteins by fractionation or by using an iTRAQ or TMT labelling strategy combined with fractionation as a good compromise between high number of samples and fractionation.

### Time-of-day dependent changes in detectable genes

**Fig 1:**
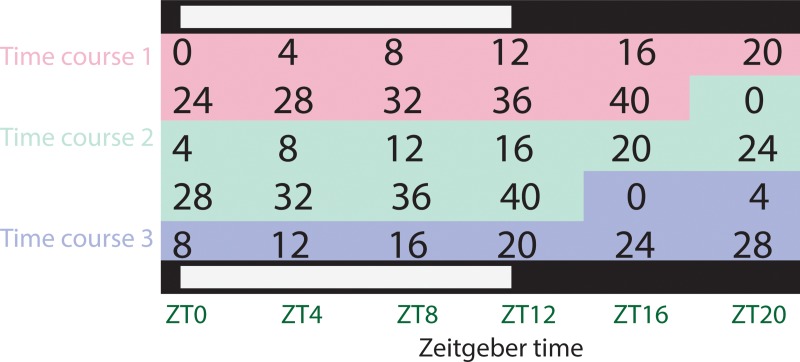
Sampling protocol for time-of-day assays. Three separate time courses were performed to achieve five biological replicates per time-of-day. Black numbers indicate the elapsed hours of the time course. Individual collections (points) and mean (red line) values are provided. The horizontal white/black bar provides represents the light/dark (*i*.*e*. day/night) conditions that the mosquitoes were collected under.

In order to determine if there is a time-of-day dependent ability to detect proteins, mosquito bodies were collected every four hours from three staggered time courses (Fig 1) of between 28–44 hr (Fig 1). Note in this collection protocol, each of five times-of-day are sampled, independently, five times (see Fig 1). Untargeted, quantitative proteomics was performed (S2 Table, S3 Table), and a total of 1525 body proteins were deemed quantifiable (identified with a least two component peptides) from the body samples.

**Fig 2:**
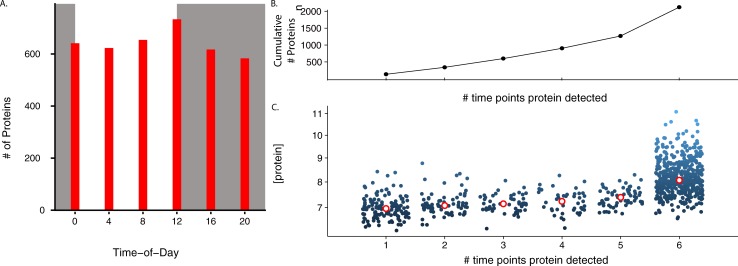
Mosquitoes samples were collected every four hours across the 24-hr day and quantitative proteomics performed. **A.** Histogram of the time-of-day (expressed in ZT time) of the number of proteins with peak abundance at each time point. Shading represents the light:dark cycle. No time point stands out as different from the others. **B.** Cumulative number of detectable proteins as additional time points are added. As an example, a protein only detectable at ZT20 or a protein only detectable at ZT0 is represented here as detected at one time point. A protein detected at *both* ZT20 and ZT0, however, would be represented here as being detected at two time points. **C.** Normalized protein intensity extracted from Progenesis analysis segmented by the maximum number of time points the protein was detected. Note that the proteins detected at more time points, have higher average protein concentrations. The unfilled red dots are the median value for each group. Time points where proteins were not detected were not used to compute average protein concentration across time points.

Our data revealed differences in the total number of proteins that were quantifiable at any given time-of-day (583–733 proteins per time-of-day) (Fig 2A). When the identity of proteins detectable (*i*.*e*. quantity > 0) is considered, there are a number of proteins only detectable at certain times-of-day. Whereas 489 proteins were detectable at all sampling times, there were between 45 and 72 proteins that were only detectable at 2–5 times-of-day, and 134 were only detectable at a single time-of-day (Fig 2B). Not surprisingly, proteins that had a higher average abundance were generally detectable at more times-of-day (Fig 2C).

### Identification of rhythmic genes

**Fig 3:**
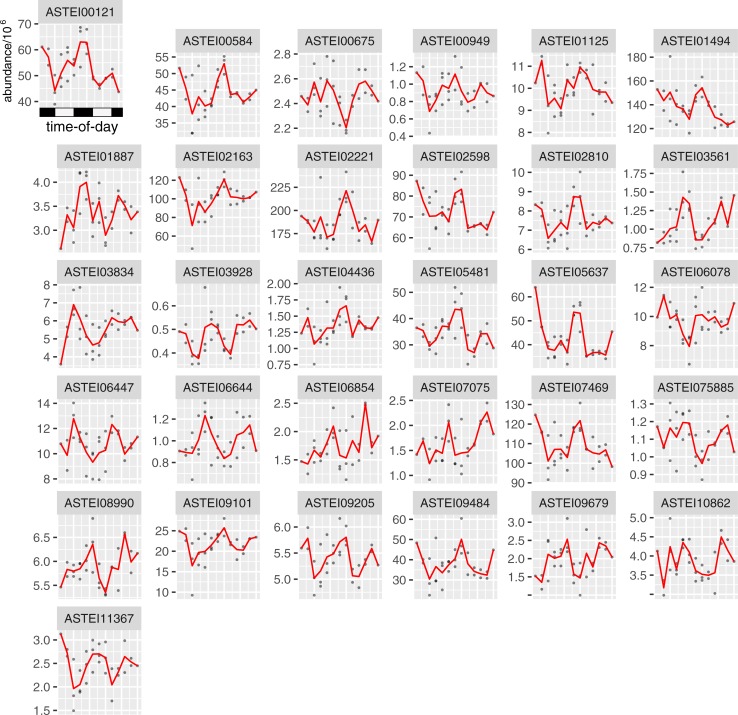
Proteins identified as rhythmic by JTK_CYCLE (*p* < 0.1, period length = 24hr). Each sub panel provides the protein abundance of one protein from three replicate experimental collections of mosquitoes. The horizontal white/black bar represents the light/dark (*i*.*e*. day/night) conditions under which the mosquitoes were collected. See Table 1 for gene name/functions.

We extended our analysis further by looking for genes that were rhythmically expressed using an algorithm (JTK_CYCLE) specifically designed for looking for “circadian” expression patterns. First, we analyzed the subset of 357 proteins that were detected at all time points, and where >1 peptide was used to identify each protein in each sample at each timepoint. Of these proteins, ANOVA revealed 90 proteins, where at least one time point was significantly different from the others (*p* < 0.1). Next, from the list of proteins with statistically significant time-of-day differences in protein concentration (90 proteins), we proceeded to analyze those proteins for 24 hr daily rhythms in abundance (rather than only a simple time-of-day difference) using the JTK_CYCLE algorithm. This algorithm is used to mine ‘omic data for such 24 hour rhythms [30, 31], and we thus applied it to our data. JTK_CYCLE identified 31 proteins as having rhythmic expression (Fig 3). As *An*. *stephensi* has not been extensively annotated, we mapped these 31 proteins to their homologues in *Ae*. *aegypti*, *An*. *gambiae*, *Culex quinquefasciatus*, and/or *D*. *melanogaster* to assign a name/function to each protein (Table 1). We note these rhythmic proteins display a wide range of phases (times-of-day when proteins peak) and abundance amplitudes (Fig 3, Table 1).

**Table 1:** Rhythmic proteins identified.

Indian-strain geneID	SDA-500 geneID	JTK_CYCLE values		Dipteran homologues **	
		ADJ.P	Phase (ZT) ***	Ensembl ID	Species: Name
ASTEI00121	ASTE003060	0.013	18	CPIJ013361	CQUI: Tropomyosin-1
ASTEI00584	ASTE004515 ASTE008899	0.018	20	AAEL010814*	AAEG: isocitrate dehydrogenase
				AGAP003167*	AGAM: NAD(P) transhydrogenase
ASTEI00675	ASTE008899	0.015	8	n/a	
ASTEI00949	ASTE002146 ASTE011453	0.090	16	AGAP003937*	AGAM: AGAP003937
				AGAP003936*	AGAM: small nuclear ribonucleoprotein D2
ASTEI01125	ASTE005445	0.030	20	CPIJ011528	CQUI: NADH dehydrogenase iron-sulfur protein 2, mitochondrial
ASTEI01494	ASTE001799 ASTE001800 ASTE001801	0.012	22	FBgn0019968	DMEL: Kinesin-73
				FBgn0001128*	DMEL: GPD-C
				AGAP007593*	AGAM: glycerol-3-phosphate dehydrogenase (NAD+)
ASTEI01887	ASTE007267	0.020	8	AAEL003211*	AAEG: beta-carotene dioxygenase
				AGAP008143*	AGAM: AGAP008143
ASTEI02163	ASTE010238	0.063	20	AGAP005558	AGAM: peptidase (mitochondrial processing) beta
ASTEI02221	ASTE009536	0.026	20	AGAP005627*	AGAM: creatine kinase
				AGAP012924*	AGAM: Arginine kinase
ASTEI02598	ASTE008307	0.056	18	AGAP006936*	AGAM: Mitochondrial cytochrome c1 heme protein
ASTEI02810	ASTE010498	0.071	18	CPIJ000098	CQUI: Electron transfer flavoprotein-ubiquinone oxidoreductase
ASTEI03561	ASTE000276	0.001	10	FBgn0263594	DMEL: RH35990p
ASTEI03834	ASTE011384	0.056	4	AAEL007698*	AAEG: PIWI
ASTEI03928	ASTE003184	0.001	14	AGAP010051*	AGAM: AGAP010051
ASTEI04436	ASTE006286	0.090	18	AGAP000720*	AGAM: Neuronal cell adhesion molecule
ASTEI05481	ASTE011491	0.044	16	CPIJ019398	CQUI: Myosin light chain 2
ASTEI05637	ASTE010982	0.009	18	AGAP011131*	AGAM: F-type H+-transporting ATPase subunit d
ASTEI06078	ASTE002102	0.050	22	AGAP012100	AGAM: 40S ribosomal protein S26
ASTEI06447	ASTE009294	0.003	2	AAEL003427	AAEG: 40S ribosomal protein S16
ASTEI06644	ASTE008693	0.008	8	AGAP008364*	AGAM: thioester-containing protein 15
ASTEI06854	ASTE001249	0.034	10	FBgn0031021	DMEL: NADH dehydrogenase (ubiquinone) 18 kDa subunit
ASTEI07075	ASTE002586	0.026	12	AGAP004055*	AGAM: 2-oxoglutarate dehydrogenase E2 component
ASTEI07469	ASTE001425	0.071	18	AAEL014913	AAEG: Pyruvate kinase
ASTEI075885	ASTE010928	0.071	10	AGAP004146*	AGAM: Ras-related protein Rab-1A
ASTEI08990	ASTE000090	0.007	10	AGAP010895*	AGAM: spectrin beta
ASTEI09101	ASTE006195	0.003	18	AGAP007841*	AGAM: F-type H+-transporting ATPase subunit delta
ASTEI09205	ASTE008202	0.023	18	AGAP011800	AGAM: Transaldolase
ASTEI09484	ASTE004410	0.034	18	AGAP010404*	AGAM: Glutathione S-transferase
				AAEL011741*	AAEG: AAEL011741
ASTEI09679	ASTE002472 ASTE002473	0.020	10	AAEL007881*	AAEG: AAEL007881
ASTEI10862	ASTE001429	0.004	10	FBgn0016693	DMEL: Putative Achaete Scute Target 1
ASTEI11367	No match	0.012	16	AGAP007122	AGAM: Tubulin, *alpha* 1

* The listed homologue has been found to be expressed rhythmically in *Aedes aegypti* [32], *Anopheles gambiae* [2], or *Drosophila melanogaster* [33]

** Only rhythmic homologues and/or a representative *named* homologue in AAEG: *Ae*. *aegypti*, AGAM: *An*. *gambiae*, CQUI: *Culex quinquefasciatus*, or DMEL: *D*. *melanogaster* is provided.

*** The calculated time-of-day, in zeitgeber (ZT) time, when protein abundance peaks where ZT0 is lights on and ZT12 is lights off.

#### Conservation of rhythmicity across species

We next searched published studies of rhythmic gene expression in *Ae*. *aegypti* [32], *An*. *gambia*e [2], and *D*. melanogaster [33] to determine if homologues of rhythmic *An*. *stephensi* protein were rhythmic in these species at the gene expression level. Indeed, we determined that of our 31 rhythmically identified *An*. *stephensi* proteins, 17 had homologues in at least one of the other three species with rhythmic expression of the same gene (Table 1). This represents ~55% of identified *An*. *stephensi* rhythmic proteins. For example, considering *An*. *stephensi* protein ASTEI01494, which is predicted to be glycerol 3-phosphate based on homology to *D*. *melanogaster* (FBgn0001128) and *An*. *gambiae* (AGAP007593), we find the *An*. *stephensi* protein abundance is rhythmic, as are the gene expression levels in *An*. *gambiae* gene expression and *D*. *melanogaster* expression levels. Similarly, protein abundance levels of ASTEI00584 and expression levels of the homologous hydrogenase genes in *Ae*. *aegypti* (AAEL010814)/*An*. *gambiae* (AGAP003167) are also both rhythmic (see Table 1).

#### Proteogenomic analysis

In order to determine if our proteomics work could be used to improve the *An*. *stephensi* genome, we next performed a proteogenomic analysis. Two sets of computed proteomes from VectorBase were utilized: (1) the *An*. *stephensi* proteome (Indian strain peptide sequences, AsteI2.3 geneset with 11,789 entries); and (2) the complete proteomes stored in VectorBase (here referred to as “All Vectors”, with >566,000 entries). “All vectors” comprises 39 proteomes (S4 Table), including other mosquito proteomes and other vectors such as snails, ticks, and kissing bugs. In other words, we analyzed the spectra generated from each peptide and compared it against both a list of *An*. *stephensi* computed peptide spectra and our “All Vectors” computed peptide spectra. A spectrum that was found in “All Vectors” but not *An*. *stephensi* was deemed to be missing from the *An*. *stephensi* genome, since we had proteomic evidence of the existence of the peptide as computed from other species (of varying degrees of relatedness).

There were 57,726 peptide matches between our data and the *An*. *stephensi* (Indian strain) proteome in VectorBase. Using a score cut-off based on identity score (average of 35.6 for “All Vectors” and 19 for *An*. *stephensi*) the number of retained high confident peptides matches decreased to 82,402 and 32,949 for “All Vectors” and *An*. *stephensi*, respectively. We manually validated all hits which were found in both sets and removed any peptides that had identical or nearly identical scores in both “All Vectors” and *An*. *stephensi* (*e*.*g*. isoleucine to leucine permutations are isobaric and are thus not easily distinguishable by mass spectrometry).

**Fig 4:**
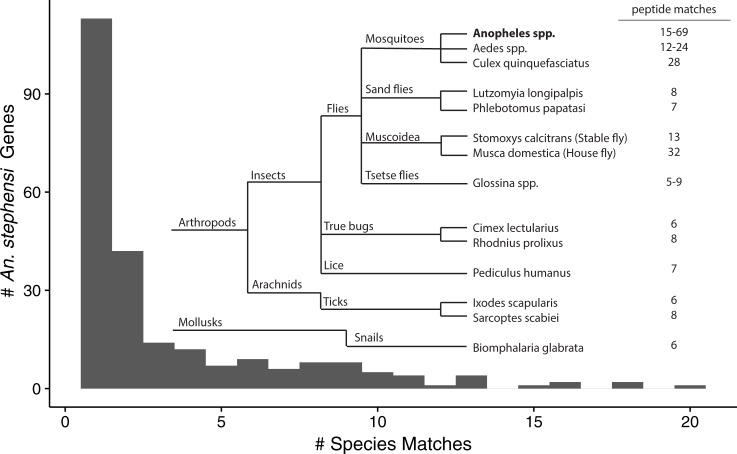
*An*. *stephensi* proteogenomic analysis revealed evidence for the presence of *An*. *stephensi* peptide sequences that are not in the predicted *An*. *stephensi* (Indian strain) proteome but match predicted peptide sequences from species across a wide-range of taxa. Numbers listed as matches represent the number of ‘missing’ *An*. *stephensi* peptide groups found in the given taxonomic group. *Anopheles* species excludes the *An*. *stephensi* (SDA-500) strain.

From the preceding analyses and filtering (such as excluding matches to SDA-500, another *An*. *stephensi* strain) we identified a total of 792 (S5 Table) high-confidence matches between peptide sequences that are ‘missing’ from the *An*. *stephensi* (Indian strain) proteome, yet are found in the 39 “All Vectors” (S4 Table) proteomes. Some identified ‘missing’ peptides had only a match in another single organism, while other peptides could be found in up to 35 other proteomes. By also combining peptide sequences that are completely contained within longer peptide sequences that were also detected, we are left with 239 unique peptide groups (*i*.*e*. ATAQLIESIK, ATAQLIE, and ATAQL count as one peptide group) that were detected using proteomics in *An*. *stephensi* samples, that matched at least one peptide sequence in one of the 39 other proteomes, but are not found in the currently available predicted Indian strain *An*. *stephensi* proteome. Matches were found across a wide phylogenic diversity, from lice to snails–not just in other mosquitos or diptera (Supplemental 5, Fig 4). These matches suggest possible genome annotation errors, which we next analyzed.

#### Identification of potential errors in the genome annotations

Further analysis of the peptides detected in our study, but missing from the *An*. *stephensi* proteome (Indian strain, Astel2.3), revealed potential errors in the genome annotations and/or assembly (S6 Table). Of the 239 peptides missing from the *An*. *stephensi*, but with matches found in “All Vectors,” were 2 peptides with 100% identity to a transcript based on tBLASTn analysis. These were found to be in a different reading frame than the annotated transcript and represent missing gene models where two genes overlap—a common phenomenon in eukaryotic organisms [34]. tBLASTn analysis revealed 25 peptides with 100% identity to a genome scaffold but not to a transcript. These are most likely genes that were not called by the gene prediction software and are missing from the current geneset (Astel2.3). There were 94 peptides that had high homology to a genomic region that contained a SNP or a mismatch causing a frameshift mutation, which could be either mutations or sequencing errors. Finally, 120 peptides were not found in the genome using standard BLAST tools and may represent gaps in the genome assembly.

## Conclusions

In this work we performed untargeted quantitative proteomics on *An*. *stephensi* mosquito samples to answer three different questions: (1) Are there qualitative, time-of-day differences in the peptides? (2) What proteins can be detected as rhythmic in a 24 hour “circadian” manner? and (3) Can the *An*. *stephensi* genome be improved using proteomic data compared against genomes of other species?

By collecting mosquito samples every four hours across the day, we determined that there are time-of-day differences in the number and quantity of proteins that are detectable at any given time-of-day. Previous work in *An*. *gambiae* revealed dusk (ZT12) was the time-of-day that had the greatest number of rhythmic genes which had their peak in expression [2]. It was hypothesized that this is due to the massive change in mosquito behavior and physiology as it goes from a resting state during the day to an active, host seeking mosquito at night [2]. Congruent with this, the greatest number of detectable proteins were detected at dusk in *An*. *stephensi*. Not surprisingly, proteins that had a higher protein abundance were detectable at more times-of-the day. A total of 134 proteins were detected only at one time-of-day, and 489 were detectable at all times-of-day.

We next extended our analysis from a question of detectability at different times-of-day, to see if 24-hour (“circadian”) levels of protein abundance could be detected in our dataset. Previous work in the *An*. *gambiae* mosquito utilized targeted quantitative proteomics, whereby the protein abundance of proteins from an *a priori* list of genes of interest [3, 17] was quantified. Here we attempted to use a non-hypothesis driven, untargeted proteomic approach to quantify proteins in mosquito tissue. Work in other species has previously revealed ~20% of *An*. *gambiae* genes and at least 8% *Ae*. *aegytpi* genes are rhythmically expressed over the 24-hour day [2].

Here, in *An*. *stephensi*, we were able to detect rhythmic protein abundance levels in only 31 of the 1525 quantifiable proteins (2%). This is much lower than we expected from gene expression data from other mosquito species, but likely represents an underestimation of the true number of rhythmic proteins. One explanation for undetected rhythmic protein abundance is that low-concentration protein time courses were removed prior to analysis. These peptides may have represented protein abundance levels that were rhythmic, but dropped below the detection limits in our experimental runs at certain times-of-day. To our knowledge, this is the first untargeted quantitative proteomics performed in a mosquito species, and we reveal it can be used to reliably quantify a large number of proteins. We note, however, that some proteins may be rhythmic, or only appear at certain times-of-day, and this point should be considered when doing experimental design.

When we considered the homology of proteins here found to have rhythmic abundance levels, ~55% of identified *An*. *stephensi* rhythmic proteins have homologues in other species that have been determined, at the gene expression level, to be also rhythmic. This provides further evidence of the rhythmic nature of biological processes being conserved across species [35].

Finally, the present study revealed a number of potential errors in the current *An*. *stephensi* genome annotations and/or assembly. Untargeted proteomics could be leveraged to improve current genome annotations; however, proteomic reducibility, speed, and whole-proteome coverage are limited using our current technologies.

## Materials and methods

### Biological material

A lab colony of *An*. *stephensi* mosquitoes were maintained at ~60% relative humidity and 26°C on a 12 hr/12 hr LD cycle [11 hr full light, 11 hr darkness (0 lux) and 1 hr dawn and 1 hr dusk transitions]. Access to 8% (*w*/*v)* fructose was provided *ad libitum*. In three replicate time courses (with slightly different durations depending on available number of mosquitoes per batch, see Fig 1), mosquitoes were placed in individual containers (pots) and allowed to acclimate for several days. A pot of mosquitoes was euthanized on dry ice every four hours and placed in -80°C prior to tissue preparation. Heads were separated on dry ice from bodies (legs and wings were removed). Herein “body” describes the body of the mosquito with no head, wings, or legs.

### Sample preparation for the time series analysis

A pool of 10 bodies was used per sample, solubilized in 250ul 8M urea 1% SDS and homogenized using a Precellys cell homogenizer (Bertin Instruments). The homogenization step comprises three steps of 40s at 5000rpm with a 10s pause; the overall procedure was repeated twice. A protein assay was performed using Pierce BCA protein assay kit, and a 50μg protein equivalent was used for SDS-PAGE analysis. Samples were briefly run on SDS-PAGE gel for 10 min, extracted and digested using Shevchenko’s method [36]. Peptide extracts were then cleaned on SPE reverse phase Bond Elut LMS (Agilent). The samples were dried under low pressure (Speedvac from Thermo-Fisher) and stored at -20°C.

### HPLC-MS analysis

The dried peptide samples were re-suspended in resuspension buffer (0.05%v/v trifluoroacetic acid in water) to a final concentration of 1 μg/μl. These samples were filtered using a Millex filter before subjecting to HPLC-MS analysis. Nano-HPLC-MS/MS analysis was performed using an on-line system consisting of a nano-pump (Dionex Ultimate 3000, Thermo-Fisher, UK) coupled to a QExactive instrument (Thermo-Fisher, UK) with a pre-column of 300 μm x 5 mm (Acclaim Pepmap, 5 μm particle size) connected to a column of 75 μm x 50 cm (Acclaim Pepmap, 3 μm particle size). Samples were analyzed on a 90 min gradient in data dependent analysis (1 survey scan at 70k resolution followed by the top 10 MS/MS).

### Proteomics, protein identification and quantification

Data from MS/MS spectra were searched using MASCOT Versions 2.4 (Matrix Science Ltd, UK) against *An*. *stephensi* (Indian strain AsteI2.3) data stored in VectorBase [37, 38]. Search parameters included a maximum missed-cut value set to 2. The following features were used in all searches: i) variable methionine oxidation, ii) fixed cysteine carbamidomethylation, iii) precursor mass tolerance of 10 ppm, iv) MS/MS tolerance of 0.05 amu, v) significance threshold (p) below 0.05 (MudPIT scoring) and vi) final Mascot peptide score of 20. A complete dataset was analysed using MaxQuant v1.5.2.8 [39] assuming a Maxquant score of 45 and more.

For the time series quantification analysis Progenesis (version 4, Nonlinear Dynamics) was used for LC-MS label-free quantitation (S2 Table). Progenesis QI for proteomics software has been designed specifically to perform label-free quantitation and is capable of analyzing significant numbers of large data files due to its peak-modelling algorithm which reduces the data files by an order of magnitude without losing any information. This allows for the analysis of large data sets including large numbers of replicates that would otherwise be impractical to run. The software is enabled with a graphical user interface which allows MS data to be viewed in either two or three dimensions. This can help to verify if features have been quantified accurately. In brief, the basic software steps are as follows: (1) Alignment of runs to compensate for LC separation “between-run” variation, allowing like-for-like comparison of peptide signals; (2) Feature detection and quantitation using peak area method; (3) Peptide identification using the mascot search engine; and (4) Peptide/protein quantitation using the calculated abundance of the features to which identifications have been matched.

Only MS/MS peaks with a charge of 2+, 3+ or 4+ were considered for the total number of ‘Features’ (signal at one particular retention time and *m*/*z*) and only the five most intense spectra per ‘Feature’ were included. Normalization was first performed based on the median of the ion intensities of these sets of multi-charged ions (2+, 3+, and 4+). The associated unique peptide ion intensities for a specific protein were then summed to generate an abundance value, which was transformed using an ArcSinH function (a log transform is not ideal considering the significant amount of near zero measurements generated by the current method of detection). Based on the abundance values, within group means were calculated and from there the fold changes (in comparison to control) were evaluated. One-way ANOVA was used to calculate the *p*-value based on the transformed values. A larger dataset with samples from both heads and bodies were analyzed using Maxquant to generate a list of identified protein and peptides (S3 Table). False Discovery Rate (FDR) information is provided in Table 2.

**Table 2:** False Discovery Rate (FDR) information.

	*An*. *stephensi*	Decoy	FDR
Peptide matches above identity threshold	11867	160	1.35%
Peptide matches above homology or identity threshold	13300	248	1.86%

### Homologue identification

To generate the gene names and orthologues in Table 1, the list of strain Indian (ASTEI) target proteins detected in the time course proteomics analysis translations was matched to strain SDA-500 (ASTE) homologues. Using this translation table, Indian strain proteins were matched to dipteran orthologous genes in OrthoDB [40, 41]. (ftp://cegg.unige.ch/OrthoDB8/Eukaryotes/Genes_to_Ogs/ODB8_EukOGs_genes_ALL_levels.txt.gz). Filtering was performed in a Jupyter notebook [42] using the Pandas (v0.18.1) Python library [43]. The notebook (https://figshare.com/s/dbb89cb869f416979f60) is accessible on FigShare. Some gene names were manually supplemented using VectorBase [37, 38] or FlyBase [44].

### Statistical analysis for rhythmic genes

In order to detect rhythmic protein abundance, we first only considered proteins where ANOVA revealed at least one time point is significantly different from the others (*p* < 0.1). Abundance data for those proteins was then processed with JTK_CYCLE [30] using the MetaCycle R package [31] to identify rhythmic proteins with a 24 hr period. Proteins were called rhythmic when their quantified protein abundance was determined by rhythmic by JTK_CYCLE (*p* < 0.1). We report at *p* = 0.09, *q* = 0.20 as our false discovery rate.

### Proteogenomic analysis

A proteogenomic analysis was performed using the Mascot (Matrix science) package. Two sets of computed proteomes from VectorBase [37, 38] were utilized: (1) the *An*. *stephensi* proteome (Indian strain peptide sequences, AsteI2.3 geneset with 11,789 entries); and (2) the entirety of arthropod proteomes stored in VectorBase (“All Vectors”, with >566,000 entries). This comprises 39 proteomes (S4 Table), both a second *An*. *stephensi* strain (SDA-500), other mosquito proteomes, snails, and other arthropod vectors such as sandflies, ticks, and kissing bugs.

We generated a subset of MS/MS features by removing MS/MS feature redundancy (keeping a maximum of the 5 most abundant peaks having the same masses and retention time). The merge .mgf file was generated using Progenesis. The dataset was searched against: (1) the *An*. *stephensi* proteome stored in VectorBase; and (2) against all the proteomes stored in VectorBase. We filter the identified peptide as follow: we only kept in both searches peptides having a Mascot score above identity and were ranked as first hit. We compared the two datasets and removed any specific peptide having the same score in both databases or showing similar peptide permutations such as isoleucine to leucine, which have indistinguishable mass spectrometry readings.

## Supporting information

S1 Table Identified peptides and proteins from untargeted proteomics. (XLSX)

S2 Table Progenesis output. (XLSX)

S3 Table Results of quantitative proteomics of *An. stephensi* bodies. (XLSX)

S4 Table List of reference species and gene build versions from VectorBase used for proteogenomic analyses. (DOCX)

S5 Table “Missing peptides” not found in *An. stephensi*, but with matches found in other species. (CSV)

S6 Table BLAST analysis of *An. stephensi* genome assembly (Astel2-Indian strain) using the peptide sequences from proteomics analysis revealed missing genome annotations. Peptide transcript hit (100%), 100% identity to *An*. *stephensi* gene transcript with full query length using tblastn; Scaffold hit (100%), 100% identity to *An*. *stephensi* genome scaffold with full query length using tblastn; Wobbly scaffold hit (<100% hit) <100% identity to *An*. *stephensi* genome scaffold with 1 mismatch and with full query length ± 1 with tblastn; No hit, no hits (not transcript, scaffold, or wobbly scaffold hit) in *An*. *stephensi* genome using tblastn, missing in genome assembly.
(CSV)
