# Supplementary material for: Genome annotation improvements from cross-phyla proteogenomics and time-of-day differences in malaria mosquito proteins using untargeted quantitative proteomics
Source: PLoS One. 2019 Jul 29;14(7):e0220225. doi: 10.1371/journal.pone.0220225 (PMC6663012; doi:10.1371/journal.pone.0220225)
Supplement: S4 Table — (DOCX) [file pone.0220225.s004.docx]

Aedes-aegypti-Liverpool AaegL3.4

Aedes-albopictus-Foshan AaloF1.2

Anopheles-albimanus-STECLA AalbS2.3

Anopheles-arabiensis-Dongola AaraD1.5

Anopheles-atroparvus-EBRO AatrE1.4

Anopheles-christyi-ACHKN1017 AchrA1.4

Anopheles-coluzzii-Mali-NIH AcolM1.4

Anopheles-culicifacies-A-37 AculA1.4

Anopheles-darlingi-Coari AdarC3.5

Anopheles-dirus-WRAIR2 AdirW1.5

Anopheles-epiroticus-Epiroticus2 AepiE1.4

Anopheles-farauti-FAR1 AfarF2.3

Anopheles-funestus-FUMOZ AfunF1.6

Anopheles-gambiae-PEST AgamP4.6

Anopheles-maculatus-maculatus3 AmacM1.4

Anopheles-melas-CM1001059_A AmelC2.4

Anopheles-merus-MAF AmerM2.4

Anopheles-minimus-MINIMUS1 AminM1.5

Anopheles-quadriannulatus-SANGWE AquaS1.6

Anopheles-sinensis-China AsinC2.2

Anopheles-sinensis-SINENSIS AsinS2.2

Anopheles-stephensi-Indian AsteI2.3

Anopheles-stephensi-SDA-500 AsteS1.5^[[1]](#footnote-1)^

Biomphalaria-glabrata-BB02 BglaB1.5

Cimex-lectularius-Harlan ClecH1.3

Culex-quinquefasciatus-Johannesburg CpipJ2.3

Glossina-austeni-TTRI GausT1.4

Glossina-brevipalpis-IAEA GbreI1.4

Glossina-fuscipes-IAEA GfusI1.4

Glossina-morsitans-Yale GmorY1.6

Glossina-pallidipes-IAEA GpalI1.4

Glossina-palpalis-IAEA GpapI1.2

Ixodes-scapularis-Wikel IscaW1.5

Lutzomyia-longipalpis-Jacobina LlonJ1.3

Musca-domestica-aabys MdomA1.3

Pediculus-humanus-USDA PhumU2.2

Phlebotomus-papatasi-Israel PpapI1.3

Rhodnius-prolixus-CDC RproC3.2

Sarcoptes-scabiei-Arlian SscaA1.2

Stomoxys-calcitrans-USDA ScalU1.2

1. Used to exclude matches from futhur analysis [↑](#footnote-ref-1)
